# Analysis of Antimicrobial Peptide Metabolome of Bacterial Endophyte Isolated From Traditionally Used Medicinal Plant *Millettia pachycarpa* Benth

**DOI:** 10.3389/fmicb.2021.656896

**Published:** 2021-06-01

**Authors:** Ng Ngashangva, Pulok Mukherjee, K. Chandradev Sharma, M. C. Kalita, Sarangthem Indira

**Affiliations:** ^1^A National Institute of Department of Biotechnology, Institute of Bioresources and Sustainable Development (IBSD), Govt. of India, Imphal, India; ^2^Department of Biotechnology, Gauhati University, Guwahati, India

**Keywords:** endophytes, genome, medicinal plant, peptides, proteomics, resistance

## Abstract

Increasing prevalence of antimicrobial resistance (AMR) has posed a major health concern worldwide, and the addition of new antimicrobial agents is diminishing due to overexploitation of plants and microbial resources. Inevitably, alternative sources and new strategies are needed to find novel biomolecules to counter AMR and pandemic circumstances. The association of plants with microorganisms is one basic natural interaction that involves the exchange of biomolecules. Such a symbiotic relationship might affect the respective bio-chemical properties and production of secondary metabolites in the host and microbes. Furthermore, the discovery of taxol and taxane from an endophytic fungus, *Taxomyces andreanae* from *Taxus wallachiana*, has stimulated much research on endophytes from medicinal plants. A gram-positive endophytic bacterium, *Paenibacillus peoriae* IBSD35, was isolated from the stem of *Millettia pachycarpa* Benth. It is a rod-shaped, motile, gram-positive, and endospore-forming bacteria. It is neutralophilic as per Joint Genome Institute’s (JGI) IMG system analysis. The plant was selected based on its ethnobotany history of traditional uses and highly insecticidal properties. Bioactive molecules were purified from *P. peoriae* IBSD35 culture broth using 70% ammonium sulfate and column chromatography techniques. The biomolecule was enriched to 151.72-fold and the yield percentage was 0.05. Peoriaerin II, a highly potent and broad-spectrum antimicrobial peptide against *Staphylococcus aureus* ATCC 25923, *Escherichia coli* ATCC 25922, and *Candida albicans* ATCC 10231 was isolated. LC-MS sequencing revealed that its N-terminal is methionine. It has four negatively charged residues (Asp + Glu) and a total number of two positively charged residues (Arg + Lys). Its molecular weight is 4,685.13 Da. It is linked to an LC-MS/MS inferred biosynthetic gene cluster with accession number A0A2S6P0H9, and blastp has shown it is 82.4% similar to fusaricidin synthetase of *Paenibacillus polymyxa* SC2. The 3D structure conformation of the BGC and AMP were predicted using SWISS MODEL homology modeling. Therefore, combining both genomic and proteomic results obtained from *P. peoriae* IBSD35, associated with *M. pachycarpa* Benth., will substantially increase the understanding of antimicrobial peptides and assist to uncover novel biological agents.

## Introduction

Antimicrobial resistance (AMR) has been developing very fast, and novel biomolecules are required to counter it as it has incurred huge loss on human life (Fair and Tor, 2014; World Health Organization, 2019). Antibiotic resistance has caused more than 2 million infections and 23,000 deaths per year in the United States, at a direct cost of $20 billion and additional productivity losses of $35 billion (WHO, 2014). In Europe, an estimated 25,000 deaths are attributable to antibiotic-resistant infections, costing €1.5 billion annually in direct and indirect costs (European Centre for Disease Prevention and Control, 2019). It is predicted that by 2050, 10 million lives a year and a cumulative US$ 100 trillion of economic output are at risk in the South-East Asian region (WHO, 2014). These facts allow us to piece together a description of AMR burden and the need of novel antimicrobial agents.

Lately, many important secondary metabolites (SMs) have been reported including anticancer taxol and taxane production by *Taxomyces andreanae*, an endophytic fungus of Pacific yew (Stierle and Strobel, 1993) and antitumor exo-polysaccharides from endophyte, *Bacillus amyloliquefaciens* sp. of the medicinal plant, *Ophiopogon japonicas* (Chen et al., 2013). These findings from medicinal plants and their associated endophytes offer a huge prospect for finding novel biomolecules (Cragg et al., 1997; Alvin et al., 2014; Li et al., 2020). Purportedly, bioprospecting the less discovered North-East India pristine forest offers a bountiful resource (Myers et al., 2000). An endophytic gram-positive bacterium, *Paenibacillus peoriae* IBSD35 was isolated from *Millettia pachycarpa* Benth. in our previous experiment using the standardized surface sterilization method (Srivastava, 2010; Ngashangva et al., 2019). Novel antimicrobial peptides (AMP) discovery requires the materials of ethnopharmacology, herbal medicines, and traditional knowledge systems to facilitate the process (Mukherjee, 2019). It was suggested that routine efforts to identify active principles from crude extracts may not be sufficient, but rather more advanced scientific research in traditional medicine to obtain evidence is required (Patwardhan, 2015).

Natural products have always played a key role in our understanding of biology and drug development (Newman and Cragg, 2012). Among them, naturally occurring peptides represent one of the first evolved and highly conserved chemical defenses of prokaryotes and eukaryotes against foreign invading pathogens (Zasloff, 2002; Boman, 2003; Arnison et al., 2013; Malmsten, 2014). They act as selective antimicrobial products of microbes in association with their host (Rosenblueth and Martínez-Romero, 2006; Hardoim et al., 2008). Genome mining in recent years has accelerated the bio-chemical workflow, and the prediction of secondary metabolites, biosynthetic gene clusters, and pathways have provided a rationale for targeted isolation of AMPs from complex protein mixtures (Harvey et al., 2015; Collins et al., 2017; Li et al., 2020). Furthermore, the ability of mass spectrometry to identify and quantify thousands of proteins from complex samples have positively affected the discovery of novel antimicrobial agents (Dancík et al., 1999; Aebersold and Mann, 2003; Junqueira et al., 2008; Cimermancic et al., 2014; Lu et al., 2014; Perkins et al., 1999).

Therefore, in this study we have reported the isolation of an antimicrobial peptide and inferred its biosynthetic gene cluster from collective analysis of both genomic and proteomic data. It will enhance the understanding of biological processes and the possibility of their application in future drug development and food preservation.

## Materials and Methods

### *Paenibacillus peoriae* IBSD35 Culture Isolated From *M. pachycarpa* Benth

*P. peoriae* IBSD35 isolated from the stem of *M. pachycarpa* Benth. (Ito et al., 2006; Srivastava, 2010) was revived from the stock (Sanyo Biomedical Freezer) preserved in the Microbial Repository Centre of IBSD (Accession No. MRC-75001) (Ngashangva et al., 2019). It was streaked on a Luria Bertani (LB) agar plate and incubated in an Eppendorf innova^*R*^42 Rotary shaker at 38°C for 12 h to check the colony purity and contamination. The colonies were sub-cultured repeatedly to obtain a pure culture (Sanders, 2012). A pure colony was picked and inoculated in a 25 ml Erlenmeyer flask (He et al., 2007).

The under-study strain physiochemical characteristics were examined by following the keys of *Bergey’s Manual of Determinative Bacteriology* and gram-staining (Holt et al., 1994). The strain motility was tested by the hanging drop method (Brock, 1999). The genomic and molecular characters were analyzed by draft genome sequencing using Illumina HiSeq 2500 (Schuster, 2008; Korostin et al., 2020) and deposited in the NCBI Genebank (Tatusova et al., 2016).

### Fermentation of *Paenibacillus peoriae* IBSD35

The overnight grown *P. peoriae* IBSD35 inoculum was seeded in a 5,000 ml Erlenmeyer flask to obtain sufficient quantities of crude extracts (He et al., 2007). The inoculum was allowed to grow until the death phase and their growth status were measured at OD∼600 (Abriouela et al., 2003). The fermentation broth was centrifuged (Centrifuge 5810 R, Eppendorf) to obtain a cell-free supernatant (CFS) (Alkotaini et al., 2013). Its retention of antimicrobial activity against *Staphylococcus aureus* ATCC 25923 was checked (Magaldi et al., 2004; CLSI, 2006, 2008). Calcium carbonate and a unit of catalase were added to impede acid production and catalytic enzymes. Toluene was added to avoid contamination from other microbes (He et al., 2007).

### Purification of AMPs From Fermentation Broth

The CFS was enriched with slow addition of ammonium sulfate; the suspension was stirred in a magnetic shaker (Tarsons Spinot Digital MC 02) for 4 h (Krisna and Sandra, 2014). The precipitate was harvested by centrifugation, while the supernatant was discarded which devoided antimicrobial activity. The precipitate was resuspended in distilled water and desalted. The sample was further purified with diethylaminoethyl cellulose (DEAE-C) column chromatography (Mahyhew and Howell, 1971), dialyzed, and purified in RP-HPLC UFLC CBM-20A (Shimadzu, Tokyo, Japan) (Herraiz, 1997). The sample was loaded on a semi-preparative Agilent ZORBAX 300SB reverse-phase C-18 of 5 μm and a 9.4 X 250 mm column and eluted out with 0.1% ion pairing reagent and trifluoroacetic acid (TFA) (HPLC grade) (Conlon, 2006). The peaks were pooled together and lyophilized in a Modulyod Freeze Dryer (Thermo) to form powder (35XT) (Ngashangva et al., 2019). The retention of antimicrobial activity was tested at each step of purification (Bradford, 1976; Magaldi et al., 2004; CLSI, 2008). Its total protein, yield percentage, and purification fold were calculated using UV-spectrometry as compared to the initial starting cell-free supernatant (Simonian, 2004). Its antimicrobial activity spectrum was tested against *S. aureus* ATCC 25923, *Escherichia coli* ATCC 25922, and *Candida albicans* ATCC 20231 (Magaldi et al., 2004; CLSI, 2006, 2008). The protein mixture was analyzed through liquid chromatography-mass spectroscopy (LC-MS) (Aebersold and Mann, 2003).

### Mass Spectrometric Analysis of Peptide Mixtures

A total of 50 μl of the RP-HPLC purified sample (35XT) was reduced with 5 mM of Tris (2-carboxyethyl) phosphine hydrochloride (TCEP), and alkylated with 50 mM of iodoacetamide, and then digested with trypsin (Sonia and Jata, 2020). Digests were cleaned using a C_18_ silica cartridge and dried using a speed vacuum. The dried pellet was resuspended in buffer A (5% acetonitrile, 0.1% formic acid). The experiment was performed using the EASY-nLC 1000 system (Thermo Fisher Scientific) coupled to a Q Exactive mass spectrometer (Thermo Fisher Scientific) equipped with a nano-electrospray ion source (Michalski et al., 2011). The fraction was digested with trypsin (1:50, trypsin/lysate ratio) for 16 h at 37°C. A total of 1.0 μg of the peptide mixture was resolved using a 15 cm PicoFrit column (360 μm outer diameter, 75 μm inner diameter, 10 μm tip) filled with 1.9 μm of C18-resin (Dr. Maeisch, Germany) (Sonia and Jata, 2020). The peptides (VP_1369) were loaded with buffer A (5% acetonitrile, 0.1% formic acid) and eluted with a 0–40% gradient of buffer B (95% acetonitrile, 0.1% formic acid) at a flow rate of 300 nl/min for 90 min. Liquid chromatography coupled to tandem mass spectrometry (LC/MS/MS) was used to identify the components of extracellular protein complex (Seidler et al., 2010; Lu et al., 2014). MS data were acquired using a data-dependent top 10 method dynamically choosing the most abundant precursor ions from the survey scan.

### Proteomics Data Processing

Samples (VP_1369) were processed and one generated RAW file was analyzed with Proteome Discoverer (Wang et al., 2008) against the *P. peoriae* IBSD35 Uniprot reference Proteome database (Table 2; Apweiler et al., 2004). For the SEQUEST search, the precursor and fragment mass tolerances were set at 10 ppm and 0.5 Da, respectively (Tabb et al., 2001; Katz et al., 2010). The protease used to generate peptides, i.e., enzyme specificity was set for trypsin/P (cleavage at the C terminus of “K/R”: unless followed by “P”) along with a maximum missed cleavages value of 2. Carbamidomethyl on cysteine as fixed modification and oxidation of methionine and N-terminal acetylation were considered as variable modifications for the database search. Both peptide spectrum match (PSM) and protein false discovery rate (FDR) were set to 0.01 FDR (Tabb et al., 2001).

### Insights Into Biosynthetic Gene Clusters and LC-MS Proteomics Data

MS-based proteomics SEQUEST was used for the identification of proteins via database-supported interpretation of MS data (Tabb et al., 2001). *De novo* sequencing inferred protein group lists and identified the potential AMPs from MS/MS data (Dancík et al., 1999; Waridel et al., 2007; Ma and Johnson, 2012). Peptides identified from tandem mass spectrometry (MS/MS) data were analyzed with proteomics tools to classify the AMPs on the basis of physiochemical characteristics (Gasteiger et al., 2005; Wang et al., 2016). *P. peoriae* IBSD35 genome BGCs were analyzed using the NCBI database and JGI/IMG web server (Markowitz et al., 2008; Tatusova et al., 2016). IMG/ABC using AntiSMASH v 5.0 tools predicted the BGCs, and it was correlated with the protein lists predicted from the Proteomic SEQUEST database search (Tabb et al., 2001; Cimermancic et al., 2014; Hadjithomas et al., 2015; Blin et al., 2019; Krishnaveni et al., 2020). AMPs sequenced were analyzed using the ExPASy-ProtParam tool (Gasteiger et al., 2005). The 3D conformation model was generated using SWISS-MODEL homology modeling and deposited in ModelArchive (Schwede et al., 2009; Beaufays et al., 2012; Waterhouse et al., 2018).

## Results

### *Paenibacillus peoriae* IBSD35 Culture Isolated From *M. pachycarpa* Benth

**FIGURE 1:**
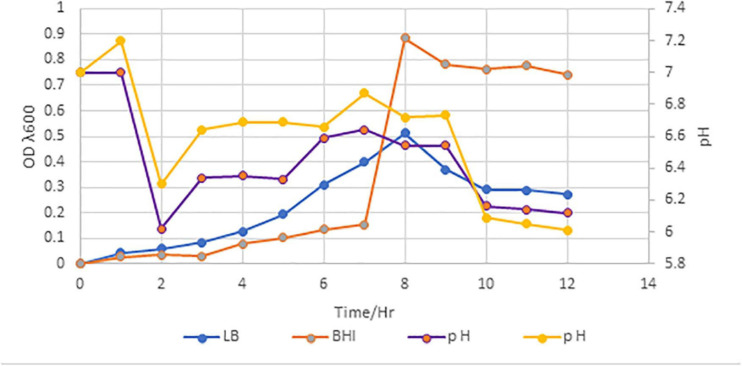
Growth curve of *P. peoriae* IBSD35 in BHI and LB broth media, and their OD and pH after 12 h incubation. A customize graph with three axes; a bottom X axis, one left Y axis and right Y axis. The X axis represents the time, and the left Y axis represents the OD. The right Y axis represents the pH (LB, Luria Bertani; BHI, brain heart infusion; OD, optical density).

*Paenibacillus peoriae* IBSD35 isolated from the stem of *M. pachycarpa* Benth. was revived by thawing for approximately 2 min using gentle agitation in a circulating water bath (Precision, Thermo Scientific) set at 25°C. Once the vial thawed, 0.5 ml of the vial was spread on the LB agar plate. The colony was picked and sub-cultured repeatedly on the LB agar plate to obtain a pure colony. A pure colony was inoculated in 25 ml of BHI broth. The culture optimum temperature and pH was found to be 6.8 and 38°C, respectively. Its antimicrobial active stage coincided with the log phase on day 6, and its optical density (OD) at λ600 was measured to be 0.9 (Figure 1). It retained antimicrobial activity against *S. aureus* ATCC 25923. Calcium carbonate (0.6%) acted as a buffer, and facilitated the reproducibility of the fermentation. A unit (10 mg ml^–1^) of catalase (0.01%) inhibited the false positive result from acid production and catalytic enzymes. Toluene (0.2%) controlled the contamination from other microbes in the CFS.

**FIGURE 2:**
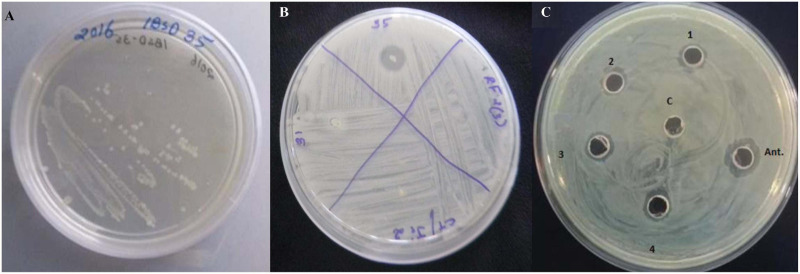
**(A)** A pure colony streak of *Paenibacillus peoriae* IBSD35 on LB agar, **(B)** Preliminary antimicrobial susceptibility test of the endophyte strains against S. aureus ATCC 25923 (P. peoriae IBSD35, strain IBSD31, strain IBSD21, and strain IBSD 22), **(C)** retention of antimicrobial activity at different stages of purification against *S. aureus* ATCC 25923 using a cut agar well diffusion bioassay (1, crude; 2, ammonium sulfate precipitate; 3, DEAE-C; 4, RP-HPLC; C, control (LB medium); and Ant., is peptide antibiotics, nisin).

The bacterial strain IBSD35 appeared whitish in color, was formed of a shiny texture, had sticky colonies, emanated a strong smell, and grew vigorously between pH 5–8.5 at a temperature range of 20–50°C on simple formulated LB medium Figure 2A. It is a slow-growing, branch-forming bacteria, it is gram-positive as it retained the pink color of the counter staining dye, safranin. It is a rod-shaped and motile bacteria. The optimum growth was observed at pH 6–7 and 38°C in BHI medium. The genomic DNA was extracted after 24 h culture and the draft genome was sequenced using the Illumina 2,500 platform. The JGI/IMG and RAST tools revealed it to be an aerobic, spore-forming, and neutrophilic bacteria. The data from this whole genome project were submitted to the EMBL/GenBank/DDBJ databases under BioProject; PRJNA434168 and BioSample; SAMN08537703 and the GenBank Accession Number PTJM01000000. Its chromosome topology is a relaxed circular DNA.

### Enrichment of the Protein Mixture From Fermentation Broth

**FIGURE 3:**
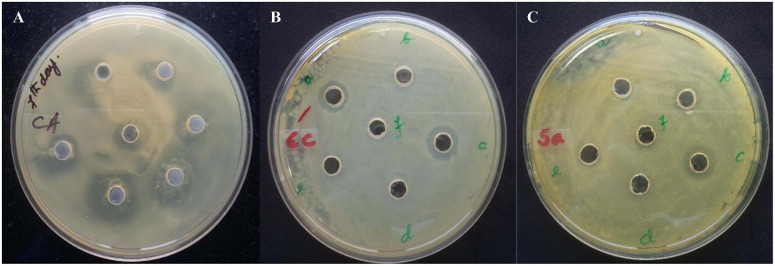
The antimicrobial agent loaded in the well diffused in the agar medium and inhibited the growth of **(A)** *S. aureus* ATCC 25923, **(B)** *Escherichia coli* ATCC 25922, and **(C)** *Candida albicans* ATCC 20231. It has shown a broad spectrum of antimicrobial activity against gram-positive, gram-negative, and fungal pathogens.

The fermentation broth was centrifuged at 4,000 rpm, 4°C for 15 min, the supernatant was recovered as CFS. Its antimicrobial activity was checked against *S. aureus* ATCC 25923 using a broth dilution bioassay. The CFS was precipitated in 70% ammonium sulfate at room temperature (25°C), the precipitate was harvested by centrifugation at 4,000 rpm, 4°C for 35 min in a 50 ml falcon tube. The pellet was resuspended in 30 ml of sterile distilled water (H_2_O). The putative protein mixture was subsequently enriched by desalting and dialysis with a 12.4 KDa molecular weight cut off (MWCO) dialysis tube (Sigma) for 12 h in 1.2 L with intermittent changing of 0.3 L of phosphate buffer saline (PBS). The dialysate was redissolved in distill. H_2_O and filtered and sterilized in 0.2 μm of Avixa. The column was irrigated with 0.1% TFA in 20% acetonitrile for 45 min at an isocratic flow rate of 2.5 ml min^–1^. The separation was monitored at 280, 205, and 214 nm and their threshold OD were recorded to be 0.083, 0.320, and 0.136, respectively. The peaks were pooled together and lyophilized to powder form (35XT), and shown to retain the antimicrobial activity. The peak (P4) retention time was 12.083 min with a purity index of 1.0000 (Supplementary Table 1). The antimicrobial bioassay against *S. aureus* ATCC 25923 at each step of purification confirmed the retention of antimicrobial activity (Figure 2B,C). The intensity of antimicrobial activity changed at different steps. The antimicrobial agent loaded in the well diffused in the agar medium and inhibited the growth of *S. aureus* ATCC 25923, *Escherichia coli* ATCC 25922, and *Candida albicans* ATCC 20231. Therefore, it has shown a broad spectrum of antimicrobial activity against gram-positive, gram-negative, and fungal pathogens (Figure 3A–C). Its total protein, yield percentage, and purification fold were calculated using UV-spectrometry as compared to the initial starting cell-free supernatant (Table 1).

**TABLE 1:** Purification scheme of the antimicrobial protein from *P. peoriae* IBSD35 culture broth (pH∼6.8 at 38°C).

**Steps**	**Vol (ml)**	**Total protein (mg)**	**Purification fold**	**Yield (%)**
CFS	2,000	880	1	100
70%(NH4)_2_SO_4_	30	14.40	1.83	3
DEAE-C	30	15	1.76	3
RP-HPLC	1	0.0029	151.72	0.05

*Its total protein, yield percentage, and purification fold were calculated as compared to the initial starting cell-free supernatant.*

### Mass Spectrometric Analysis of Peptide Mixtures

SEQUEST was used to catalog the peptide components of the complex protein mixture (VP_1369) to identify the proteins enriched from the fermentation broth of *P. peoriae* IBSD35. SEQUEST identified 15 protein groups from the partially purified complex protein mixture (Supplementary Table 2). The known genome proteins or peptides were searched with SEQUEST and PEAKS from the uninterpreted experimental MS/MS database to confirm the prediction of AMPs (Eng et al., 1994; Ma et al., 2003; Lopez-Ferrer et al., 2004). All tandem mass spectra were searched by using the SEQUEST program against the *P. peoriae* IBSD35 proteome database (Hunt et al., 1986; Tabb et al., 2001). Each high-scoring peptide sequence was manually compared with the corresponding tandem mass spectrum to ensure the match was correct (Table 2).

**TABLE 2:** LC-MS analysis of the sample (VP_1369) using the SEQUEST search algorithm against the *Paenibacillus peoriae* IBSD35 Uniprot database.

**S. no**	**Sample name**	**Sample ID**	**Database search**	**Protein groups**	**Peptides**	**Proteins**
1	35XT	VP_1369	*Paenibacillus peoriae* IBSD35	15	29	15

*MS data were acquired using a data-dependent top 10 method dynamically choosing the most abundant precursor ions from the survey scan.*

### Proteome Analysis of the *Paenibacillus peoriae* IBSD35

The AMP prediction with the CAMP_*R3*_ tool has shown that the *P. peoriae* IBSD35 genome has a 1,111 bactericidal stretch and a mean antimicrobial value of 0.25 (Waghu et al., 2015). Peptides with different molecular weight (MW) were detected from the LC-MS analysis (Table 3). The individual peptide sequences were manually analyzed using the APD3 Antimicrobial Peptide Calculator and ExPASy–ProtParam tools (Wang et al., 2016). IMG/ABC using AntiSMASH v5.0 predicted 25 BGCs from the *P. peoriae* IBSD35 genome, out of which 18 BGCs (i.e., 72%) were non-ribosomal peptide synthases (NRPSs) (Krishnaveni et al., 2020; Supplementary Figure 1). However, 13 BGCs had no similarity in the database. LC/MS analysis of the *P. peoriae* IBSD35 extract revealed that it produced Fusaricidin, Gramicidin, Bacteriocin, Andracin, Fengycin, Griselymicins, Micrococcin, Paenibacterin, Ericin, Penaeidin, Lipopeptide, Lassopeptide, Plantaricin, Lantibiotics, etc. (Table 3 and Supplementary Figure 1).

**TABLE 3:** Some of the common AMPs from LC-MS analysis of *Paenibacillus peoriae* IBSD35 culture broth using PEAKS and SEQUEST search tools.

**Peptide**	**m/z**	**z**	**RT**	**Mass**	**ppm**	**PTM**	**AMP**	**Similarity (%)**
MLMPVLHK	484.7956	2	92.32	967.5347	43.3	N	Gramicidin S	40
AGSTATAAVPVVVPLLPLEPK	1,015.567	2	105.04	2,029.182	-30.9	N	Gramicidin C	40.9
K(+42.01)NYTKC(+57.02)FKGM(+15.99)PPK	828.9275	2	88.25	1,655.816	14.5	Y	Paenibacterin	42.85
AGNVLVEGSTPPVTVHPLDK	677.3781	3	104.99	2,029.085	13.8	N	GP-19 (bacteriocin)	39.13
STC(+57.02)VTCFC(+57.02)MLVYR	820.4572	2	112.11	1,638.703	120.2	Y	Micrococcin P1	43.75
SC(+57.02)SCKPPVLVPLMMR	859.4999	2	101.03	1,716.855	76	Y	Griselimycin	40
STKAAWDMFTAPSVLWMSGTRKSSHGK	742.631	4	113.98	2,966.453	14.1	N	Lantibiotic, type 2	38.46
AGNSGYWTGPM(+15.99)M(+15.99)M(+15.99)AAP VSTYDVVLMFCQRRHGRAHVTNTVGAAGTLSSDSR	927.4861	6	109.49	5,558.58	52.7 Y	Penaeidin-3n	32.35	
VMARKRTVPELYVR	859.4999	2	101.34	1,716.982	1.8	N	Fengycin B2	46.66
AVMKVESHLC(+57.02)STKRNRTYKLLVRC (+57.02)LLPC(+57.02)KHADHTMVWNAGK	982.4637	5	93.26	4,907.523	-49	Y	Ericin A (Lantibiotic)	32.55
ACATEYTAK	479.3219	2	112.72	956.4273	210.7	N	Fusaricidine D	40
TVGRVNSM(+15.99)AQTAEGAMNEVSSMLTR	886.0884	3	112.1	2,655.242	0.8	Y	Plantaricin DL3	34.61
AC(+57.02)TVDVHK	465.307	2	107.1	928.4437	167.5	Y	Nrps, Lipopeptide	37.5
AEGVPPEVPTGVASAYR	850.4984	2	109.81	1,698.858	73.3	N	A class 2 lasso peptide; class 1 microcins	39.13
C(+57.02)GTLVDHK	465.307	2	97.02	928.4437	167.5	Y	Andracin B	41.66

*m/z, mass to charge ratio; z, charge; PTM, post translational modification; AMP, antimicrobial peptide; RT, retention time; ppm, parts per million.*

### Insights Into Biosynthetic Gene Clusters From Whole Genome and LC-MS Proteomic Data

LC-MS predicted a protein NRPS with accession number A0A2S6P0H9 and its gene is C5G87_06145 (Supplementary Table 2). It is a 3,737 aa with an MW of 41.9064 KDa. Its sequence is derived from the EMBL/GenBank/DDBJ whole genome shotgun (WGS) entry of *P. peoriae* IBSD35 which are preliminary data with accession number PPQ4949.1. It is derived by automated computational analysis using gene Protein Homology prediction (States et al., 1991; Stephen et al., 2005). Its DNA coordinates are 3–8,873 (+) (8,871 bp) with a GC content of 0.52. The NRPS BGC was blastp (blastp BLASTP 2.9.0+) using matrix Blosum62 at a threshold of 10 which showed that it was 82.4% similar to fusaricidin synthetase (E3EJA7) of *Paenibacillus polymyxa* SC2 (E-value: 0.0, score: 15,836, query length: 3,737, and match length: 3,748) (Stephen et al., 2005). A fusaricidin synthase biosynthetic gene cluster was predicted based on the evidence of CDS [Condensation and (AMP-binding or A-OX)] or (Condensation and AMP-binding).

AntiSMASH v5.0 predicted that the protein list from LC-MS data with accession no. A0A2S6P0H9 is an NRPS, and 60% of genes showed similarity to paenibacterin. Its location is 1–47,371 nucleotide with a total nucleotide of 47,371. This BGC is linked to a cluster ID 2816336711.Ga0347712_134.region1 of *P. peoriae* IBSD35 which is an NRPS with a gene count of 1 and 4 Pfam (Protein family) count (Supplementary Table 3). It is 8,875 bp long. The putative NRPS gene cluster was analyzed *in silico* which showed 37.79% sequence similarity to linear gramicidin synthase subunit A (Zheng et al., 2000; Reimer et al., 2019). Its structure assessed with a general Ramachandran plot has shown that more than 90% are inside the inner favored position (Supplementary Figure 2; Chen et al., 2010). Its 3D conformation model was generated from the SWISS-MODEL template library searched with BLAST and HHBlits (Camacho et al., 2009; Steinegger et al., 2019) for evolutionary-related structures matching the target sequence, and it was deposited at ModelArchive with accession no. DOI Will be activated as soon as the references or the article is added which refer to this Model Archive (Supplementary Figure 3).

The LC-MS proteomic analysis protein list has shown that the antimicrobial peptide MESEDHISCLPYTNHVSRSTTVTSLNSHTYTLTFPTEISQR is linked to this NRPS with accession A0A2S6P0H9. The peptide is given a name Peoriaerin II based on its source. Physiochemical properties of Peoriaerin II analyzed using the ExPASy–ProtParam tool^1^ revealed that its N-terminal is Methionine. It has four negatively charged residues (Asp + Glu) and a total number of two positively charged residues (Arg + Lys) (Table 4). Its MW is 4,685.13 Da, and the total number of atoms is 640. The estimated half-life is >10 h (Ferguson and Smith, 2003). The 3D conformation of the Peoriaerin II was predicted using homology modeling by the SWISS MODEL and deposited in ModelArchive with accession no. ma-3rxzx DOI Will be activated as soon as the references or the article is added which refer to this ModelArchive.

**TABLE 4:** The physiochemical properties of Peoriaerin II.

**Peptide**	**No of aa**	**Aliphatic index**	**Mass**	**PTM**	**GRAVY**	**Molecular formula**	**Instability index (II)**	**Theoretical PI**
MESEDHISCLPYTNHVSR STTVTSLNSHTYTLTFPTEISQR	41	61.71	4,685.13	C	-0.588	C_20__0_H_31__2_N_5__6_O_7__0_S_2_	77.41	5.75

*The SEQUEST search algorithm was used to generate the antimicrobial sequences. m/z, mass to charge ratio; m, mass; PTM, post translational modification; aa, amino acids; C, carbamidomethyl on cysteine as fixed modification.*

**FIGURE 4:**
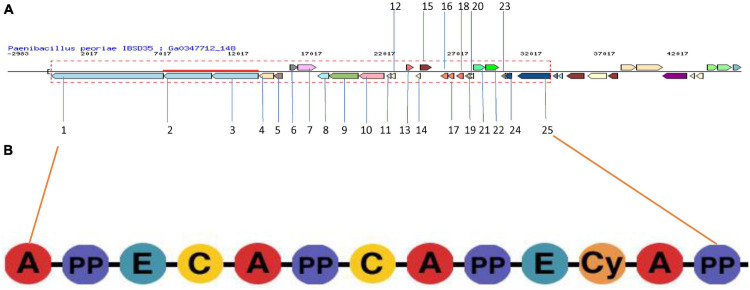
**(A)** NRPS BGC in *P. peoriae* IBSD35 Neighborhood for Cluster ID 2816336725.Ga0347712_148.region1 predicted by JGI/ABC using AntiSMASH v5.0. The genes in the red dashed box are protocore genes in the clusters for pfam00068 condensation domain functions associated with this cluster. Genes are colored by PFAM association. **(B)** Domain organization of NRPS modules. Consensus sequences of conserved motifs in NRPS and the predicted specificity of A-domainsare indicated. A, adenylation domain; PP, peptidyl carrier protein domain; and C, condensation domain.

Similar clusters were searched for in the JGI/ABC database and the heatmaps of 11 genomes were plotted in color code^2^ (Figure 4). This search and subsequent analysis have led to the identification of putative NRPS gene clusters in the selected 11 genomes (Supplementary Table 3, Blin et al., 2019; Krishnaveni et al., 2020). The BC similarity search was based on pre-calculated pairwise similarity scores using the Jaccard Index statistic for comparing two sets (Cimermancic et al., 2014; Hadjithomas et al., 2015).

**FIGURE 5:**
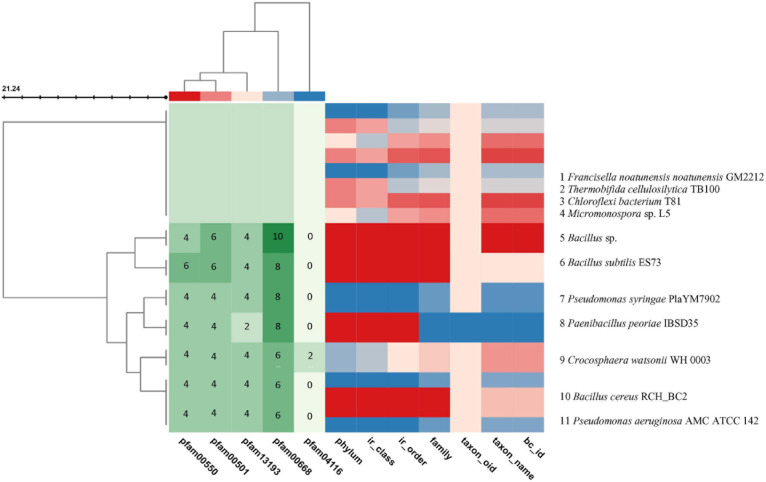
Visualization of the BGCs plotted in color code from the selected 11 genomes using the JGI/ABC database. The color code is represented in numbers to indicate the number of pfam counts using MS Word. The X axis indicates the pfam and the genome taxonomy. The genomes are indicated at the right Y axis. The left branching indicates the phylogeny relation of the BGCs. The BC similarity search was based on pre-calculated pairwise similarity scores using the Jaccard Indexstatistic for comparing two sets. The core genes are pfam00501: AMP-binding enzyme, pfam00550: phosphopantetheine attachment site, pfam00668: condensation domain, pfam04116: fatty acid hydroxylase superfamily. The list of genomes from the JGI/ABC database include: 1, *Francisella noatunensis* noatunensis GM2212; 2, *Thermobifida cellulosilytica* TB100; 3, *Chloroflexi bacterium* T81; 4, *Micromonospora* sp. L5; 5, *Bacillus* sp.; 6, *Bacillus subtilis* ES73; 7, *Pseudomonas syringae* PlaYM7902; 8, *Paenibacillus peoriae* IBSD35; 9, *Crocosphaera watsonii* WH 0003; 10, *Bacillus cereus* RCH_BC2; and 11, *Pseudomonasaeruginosa* AMC ATCC 142. (Two scores are calculated: Jaccard Score: fraction of distinct pfams shared between two BCs (intersection) over the total number of distinct pfams in both sets (union). Adjusted Jaccard Score: a modified version of the Jaccard Score that considers the similarity between the number of occurrences of each pfam in each BC).

The core genes include the phosphopantetheine attachment site (pfam00550), AMP-binding enzyme (pfam00501), AMP-binding enzyme C-terminal domain (pfam13193), and condensation domain (pfam00668) which are present in the gramicidin BGC of all the selected genomes, whereas the fatty acid hydroxylase superfamily (pfam04116) is present only in *Crocosphaera watsonii* WH 0003. A putative operon encoding the biosynthetic pathway was identified from BGC analysis (Figure 5A). Its BGC domain structure was predicted using the PKS-NRPS analysis tool, and blast results produced significant alignments to peptide synthetase I (score bits: 20, E-value: 0.010, method: compositional matrix adjust, identities: 8/8 (100%), positives: 8/8 (100%) (Marahiel et al., 1997; Brian and Jacques, 2009; Figure 5B). Visualization of the NRPS neighborhoods from the 11 genome BCs has shown that although the flanking regions of the BCs differ, the core genes are conserved, thus it is likely that these BCs indeed encode the necessary proteins.

## Discussion

AMR is spreading at an alarming rate causing innumerable public health crises. Therefore, a competent, target specific, efficient lead compound, and new therapeutic strategies are required to address this formidable challenge (Newman and Cragg, 2012). It is essential to explore new sources by intensifying screening and identifying chemical diversity equipped with cutting-edge techniques including genomics and proteomics tools (Davies, 2011; Harvey et al., 2015). The less explored region of North-East India which lies in the Indo-Burma biodiversity hotspot region (Myers et al., 2000) offered a venue to find novel biomolecule. SMs from microbes are rich sources of novel compounds, and often result from the interplay between genotypes and their immediate external environment (Rosenblueth and Martínez-Romero, 2006; Porras-Alfaro and Bayman, 2011). The study of these compounds has improved our understanding of how an organism interacts with its environment (Newman and Cragg, 2012). It is recurrently recognized that a significant number of natural product drugs or lead compounds are produced by microbes, or a microbe’s interaction with the plants and this area of research offers huge potential for finding new novel biomolecules (Friesen et al., 2011).

The study of the endophytic microorganism, *Bacillus amyloliquefaciens* sp. isolated from the medicinal plant *Ophiopogon japonicas* afforded the discovery of antitumor exo-polysaccharides derived from the genus *Bacillus* (Chen et al., 2013). Such findings from microbial endophytes provided highly promising therapeutic value for antitumor activity against gastric carcinoma cell lines. Amongst the compounds, naturally occurring peptides represent one of the first evolved chemical defenses of prokaryotes and eukaryotes against foreign invading pathogens (Zasloff, 2002; Boman, 2003). They act as selective antimicrobial products of microbes in association with their host (Rosenblueth and Martínez-Romero, 2006; Hardoim et al., 2008). AMPs’ capability of resistant development has attracted a great deal of attention (Zasloff, 2002; Yeaman and Yount, 2003; Brogden, 2005). The global peptide drug market has been predicted to increase from US$14.1 billion in 2011 to an estimated US$25.4 billion in 2018, with an underlying increase in novel, innovative peptide drugs from US$8.6 billion in 2011 (60%) to US$17.0 billion (66%) in 2018 (Transparency Market Research, 2012).

A gram-positive bacterium, *P. peoriae* IBSD35, isolated from the stem of *M. pachycarpa* Benth. retained antimicrobial activity against *S. aureus* ATCC 25923 (DeFilipps and Krupnick, 2018; Ngashangva et al., 2019). Its optimum growth condition was observed at pH∼6.8 at 38°C in LB and BHI media. It was unable to grow on highly acidic and alkaline conditions. Simple formulated medium was preferred for our extraction process because rich media stimulated biofilm formation which hindered the extraction process. The culture was harvested by centrifugation and enriched with 70% ammonium sulfate, desalted, and dialyzed. It was observed that high salt concentration impeded the further purification process. The enriched protein complex is purified with positively charged resin DEAE-C based on the biomolecule physiochemical characteristics and RP-HPLC (Conlon, 2006; Ngashangva et al., 2019). The PR-HPLC purified sample was analyzed with LC-MS/MS (Eng et al., 1994; Dancík et al., 1999). The purified sample exhibited a broad spectrum of antimicrobial activity against *S. aureus* ATCC 25923, *E. coli* ATCC 25922, and *C. ablicans* ATCC 10231, which represent gram-positive, gram-negative, and fungal pathogens. However, the potency of antimicrobial activity against pathogens at different stages of purification were different which may be due to different bioavailability. Moreover, potency toward different pathogens were different which may be due to the differences in mode of action toward pathogens’ cell membranes.

High-performance liquid chromatography enabled us to separate the complex trypsin-digested peptide mixture, and infer the protein sequence and identify hundreds of potential antimicrobial peptides from MS/MS data through *de novo* sequencing (Dancík et al., 1999; Ma et al., 2003; Waridel et al., 2007; Ma and Johnson, 2012; Medzihradszky and Chalkley, 2015). AMPs were sequenced using LC-MS which coincides with the prediction from its genome SMs BGC. The ability of mass spectrometry to identify and precisely quantify thousands of proteins from complex samples can be expected to impact broadly on biology and finding novel AMPs (Aebersold and Mann, 2003).

Protein identification is a key and essential step in the field of proteomics which can help in the classification of samples on the basis of a particular pattern. Peptide identification from tandem mass spectrometry (MS/MS) data is one of the central tasks in our experiment (Medzihradszky and Chalkley, 2015). Genome mining has accelerated the workflow, as the prediction of SM biosynthetic gene clusters and their pathways provided us with a rationale for the isolation of natural AMP from the complex protein mixture and a link to its genome BGC (McIntosh et al., 2009; Li et al., 2020).

Liquid chromatography coupled with tandem mass spectrometry (LC/MS/MS) was used to identify the components of the extracellular protein complex (Tabb et al., 2001; Seidler et al., 2010; Lu et al., 2014). MS-based proteomics were used for the identification of proteins via database-supported interpretation of MS data using search engines such as SEQUEST and PEAKS (Perkins et al., 1999; Tabb et al., 2001; Ma et al., 2003). Genome mining was used to predict the secondary metabolite biosynthetic gene clusters, and used as a rationale to link the peptides from MS/MS data (Li et al., 2020). IMG/ABC using AntiSMASH tools predicted the *P. peoriae* IBSD35 genome BGCs, and they were correlated with the protein lists predicted from the Proteomic SEQUEST database search. The sequence used blast to search for similarity and assessed their scores using the web tools of the NCBI/EMBL database (Stein et al., 2002). A NRPS BGC was predicted from LC-MS/MS data which was assumed to encode Peoriaerin II. The domain was elaborated using the NRP-PKS analyses tool from the NRPS BGC and its 3D conformation structure was assessed with a general Ramachandra plot in which more than 90% were in the inner favored position, while the Gly, Pro, and pre-Pro residues were on separate plots (not shown).

This finding is remarkable considering that the AMP is from a plant-associated bacterial endophyte. It is effective against gram-positive and gram-negative bacteria as well as fungal pathogens. This finding raises the possibility to find novel strains from traditionally used medicinal plants with novel BGC (Maroti et al., 2011). The current proteomic approach focuses on the *de novo* analysis of the protein mixture isolated from *Paenibacillus peoriae* IBSD35 fermentation broth. MS-based proteomics enabled the analysis of the extracellular metabolites and were linked with the genome sequence database (Aebersold and Mann, 2003; Wang et al., 2014). Peoriaerin II was harvested from the fermentation broth of *P. peoriae* IBSD35 by centrifugation. It showed potent antimicrobial activity against *S. aureus* ATCC 25923. MS-based proteomics is an indispensable technology to interpret the information encoded in genomes for molecular and cellular biology, and for the emerging field of system biology.

## Conclusion

Traditionally used medicinal plant, *M. pachycarpa* Benth., harbored endophytic gram-positive bacterium, *Paenibacillus peoriae* IIBSD35. The latter is a good source of novel antimicrobial peptides. It offered a new source and strategy to combat the ever-increasing menace of AMR. An antimicrobial agent which can be cultured at pH∼6.8, 38°C was harvested in an environment friendly and cost-effective experimental set up. The growth curve indicated that its log phase coincided with the antimicrobial activity state on day 6.

The extracellular metabolites harvested from the fermentation broth on log phase were enriched using 70% ammonium sulfate and desalted with a 1.24 KDa dialysis tube in 1.2 L of PBS in 12 h. High-performance liquid chromatography enabled us to separate the complex protein mixture, infer the protein sequence, and identify the potential antimicrobial peptides from MS/MS data through *de novo* sequencing. The biomolecule was enriched to 151.72-fold and the yield percentage was 0.05 which is low but it may be enhanced using supplements. The specific activity (AU/mg) increased from 1,818.18 initial crude extract to 275,862.06 RP-HPLC which indicated its increase in potency from crude to the final purified form.

It retained the antimicrobial activity at different stages of purification indicating its stability and potential for drug development and as a food preservative. Its broad spectrum of antimicrobial activity against *S. aureus* ATCC 25923, *Escherichia coli* ATCC 25922, and *Candida ablicans* ATCC 10231 indicated its efficacy against AMR pathogens. The MIC of the AMP, Peoriaerin II, against *S. aureus* ATCC 25923 was 0.0365 μg μl^–1^ which indicated its high potency.

*P. peoriae* IBSD35 genome analyses have accelerated our prediction of specific NRPS biosynthetic gene clusters and its domain organizations (Wang et al., 2014). It provided a rationale for isolation of Peoriaerin II from complex crude extracts. The proto-core gene pfam00068 condensation domain functions associated with the cluster were elaborated from its domain organization comparison in the NRPS BGC heatmap. An NRPS BGC sequence BLAST in the UniprotKB was 82.4% similar to the fusaricidin biosynthetic gene cluster of *Paenibacillus polymyxa* SC2.

Therefore, traditionally used medicinal plants from less explored forests offer new sources to find novel endophytes and their novel compounds that can be used for drug development against AMR, as food preservative, and as industrial and agricultural biological agents. Combining genomic data with the LC-MS mass spectrometry and molecular networking-based investigation of the *P. peoriae* IBSD35 metabolome, we succeeded in identifying the highly potent and broad spectrum Peoriaerin II which can be further used for drug development against AMR. Additionally, many AMPs and their variants were identified which have high potential as antimicrobial agents in medicine, agriculture, and industries.

## Data Availability Statement

The datasets presented in this study can be found in online repositories. The names of the repository/repositories and accession number(s) can be found in the article/Supplementary Material.

## Author Contributions

SI and MK conceived and designed the study. NN performed the experiments and wrote the manuscript. KS performed the RP-HPLC. SI, MK, and PM analyzed the results and data. Valerian Chem performed the LC-MS. All authors have read and approved the manuscript and reviewed and confirmed the manuscript for publication. The IBSD Manuscript No. is IBSD/2020/01/049.

## Conflict of Interest

The authors declare that the research was conducted in the absence of any commercial or financial relationships that could be construed as a potential conflict of interest.

## Footnotes

1 https://web.expasy.org/cgi-bin/protparam/protparam

2 https://img.jgi.doe.gov/cgi-bin/abc/main.cgi
