# Supplementary material for: Analysis of Antimicrobial Peptide Metabolome of Bacterial Endophyte Isolated From Traditionally Used Medicinal Plant Millettia pachycarpa Benth
Source: Front Microbiol. 2021 Jun 1;12:656896. doi: 10.3389/fmicb.2021.656896 (PMC8208310; doi:10.3389/fmicb.2021.656896)
Supplement: Supplementary file 1 [file Data_Sheet_1.docx]

**Molecular networking investigation of *Paenibacillus peoriae* IBSD35 metabolome**

**SUPPLEMENTARY**

**TABLE S1|** The peak (P4) retention time and a purity index of RP-HPLC eluents.

| **Peak** | **Ret time (Min)** | **Area** | **Height** | **Area %** | **Height %** | **Peak purity index** |
| --- | --- | --- | --- | --- | --- | --- |
| 1 | 0.224 | 3004 | 975 | 0.036 | 0.096 | 0.96835 |
| 2 | 3.760 | 6493 | 328 | 0.078 | 0.032 | 0.70032 |
| 3 | 5.830 | 587756 | 7518 | 7.034 | 0.744 | 0.96832 |
| 4 | 12.083 | 5173580 | 888359 | 61.991 | 87.882 | 1.00000 |
| 5 | 13.057 | 277854 | 14525 | 3.329 | 1.437 | 0.74329 |
| 6 | 13.664 | 511383 | 13327 | 6.127 | 1.318 | 0.92607 |
| 7 | 14.371 | 798837 | 12856 | 9.572 | 1.272 | 0.99614 |
| 8 | 15.349 | 4900 | 629 | 0.059 | 0.062 | 1.0000 |
| 9 | 16.129 | 59210 | 1502 | 0.709 | 0.149 | 0.99991 |
| 10 | 17.728 | 226291 | 14262 | 2.711 | 1.411 | 0.98275 |
| 11 | 18.270 | 5356 | 423 | 0.064 | 0.042 | 1.00000 |
| 12 | 18.776 | 628408 | 52041 | 7.530 | 5.148 | 1.00000 |
| 13 | 19.691 | 62665 | 4113 | 0.751 | 0.407 | 0.99993 |
| Total |  | 8345738 | 1010857 | 100.000 | 100.000 |  |

**TABLE S2 |** Protein group accession lists, coverage and supporting peptides from the database of *Paenibacillus peoriae* IBSD35 using LC-MS/MS.

| **Accession** | **Description** | **Score** | **Coverage** | **# Proteins** | **# Unique Peptides** | **# Peptides** | **# PSMs** | **# AAs** | **MW [kDa]** |
| --- | --- | --- | --- | --- | --- | --- | --- | --- | --- |
| A0A2S6NS86 | Copper amine oxidase | 24.53 | 18.15 | 1 | 10 | 10 | 13 | 788 | 81.7 |
| A0A2S6NS92 | Peptidoglycan-binding protein LysM | 23.06 | 21.29 | 1 | 3 | 3 | 8 | 155 | 16.5 |
| A0A0K2FDU9 | Cold shock domain-containing protein | 21.43 | 53.85 | 1 | 4 | 4 | 9 | 65 | 7.1 |
| A0A2S6NS08 | Flagellin | 5.99 | 5.06 | 1 | 1 | 1 | 2 | 257 | 27.3 |
| A0A2S6P075 | Uncharacterized protein | 2.41 | 5.35 | 1 | 1 | 1 | 1 | 243 | 26.6 |
| A0A2S6P167 | Elongation factor Tu | 1.90 | 3.28 | 1 | 1 | 1 | 1 | 396 | 43.3 |
| A0A0K2F526 | Uncharacterized protein | 0.00 | 12.15 | 1 | 1 | 1 | 1 | 181 | 21.7 |
| A0A2S6P0H9 | Non-ribosomal peptide synthetase | 0.00 | 1.10 | 1 | 1 | 1 | 1 | 3737 | 418.8 |
| A0A2S6NY96 | Cysteine ABC transporter ATP-binding protein | 0.00 | 2.24 | 1 | 1 | 1 | 1 | 715 | 79.3 |
| A0A2S6NQK5 | DNA gyrase subunit A | 0.00 | 1.76 | 1 | 1 | 1 | 1 | 853 | 95.4 |
| A0A2S6NTU2 | ATP-dependent helicase/nuclease subunit A | 0.00 | 1.94 | 1 | 1 | 1 | 1 | 1340 | 151.4 |
| A0A2S6NS95 | Protein translocase subunit SecA | 0.00 | 1.23 | 1 | 1 | 1 | 1 | 816 | 92.6 |
| A0A2S6NQ71 | Aldose 1-epimerase | 0.00 | 2.80 | 1 | 1 | 1 | 1 | 321 | 36.9 |
| A0A2S6NUT6 | Uncharacterized protein | 0.00 | 20.90 | 1 | 1 | 1 | 1 | 134 | 14.9 |
| A0A2S6NTQ9 | Polyketide beta-ketoacyl:ACP synthase | 0.00 | 11.41 | 1 | 1 | 1 | 2 | 412 | 44.9 |

**TABLE S3 |** The NRPS BGC neighbourhoods from the 11 genomes BCs acquired from similarity search in JGI/ABC database.

| **Genome Name** | **Cluster ID** | **Method** | **Pfam Count** | **Gene Count** |
| --- | --- | --- | --- | --- |
| 1 *Francisella noatunensis noatunensis* GM2212 | [2504759847.c00097_FSC775_...region1](https://img.jgi.doe.gov/cgi-bin/abc/main.cgi?section=BiosyntheticDetail&page=cluster_detail&cluster_id=2504759847.c00097_FSC775_...region1) | antiSMASH v5.0 | 4 | 1 |
| 2 *Thermobifida cellulosilytica* TB100 | [2503761410.c00530_TCel_TB...region1](https://img.jgi.doe.gov/cgi-bin/abc/main.cgi?section=BiosyntheticDetail&page=cluster_detail&cluster_id=2503761410.c00530_TCel_TB...region1) | antiSMASH v5.0 | 4 | 2 |
| 3 *Micromonospora* sp. L5 | [2501939685.c00199_Micromo...region1](https://img.jgi.doe.gov/cgi-bin/abc/main.cgi?section=BiosyntheticDetail&page=cluster_detail&cluster_id=2501939685.c00199_Micromo...region1) | antiSMASH v5.0 | 4 | 2 |
| 4 *Chloroflexi bacterium* T81 | [2503242289.c00259_T81_Chl...region1](https://img.jgi.doe.gov/cgi-bin/abc/main.cgi?section=BiosyntheticDetail&page=cluster_detail&cluster_id=2503242289.c00259_T81_Chl...region1) | antiSMASH v5.0 | 4 | 1 |
| 5 *Bacillus* sp. | [2501803077.c00142_ChemG16...region1](https://img.jgi.doe.gov/cgi-bin/abc/main.cgi?section=BiosyntheticDetail&page=cluster_detail&cluster_id=2501803077.c00142_ChemG16...region1) | antiSMASH v5.0 | 4 | 2 |
| 6 *Bacillus subtilis* ES73 | [2503755795.c00023_BSes73_...region1](https://img.jgi.doe.gov/cgi-bin/abc/main.cgi?section=BiosyntheticDetail&page=cluster_detail&cluster_id=2503755795.c00023_BSes73_...region1) | antiSMASH v5.0 | 4 | 2 |
| 7 *Pseudomonas syringae* PlaYM7902 | [2505347160.c00015_PlaYM79...region1](https://img.jgi.doe.gov/cgi-bin/abc/main.cgi?section=BiosyntheticDetail&page=cluster_detail&cluster_id=2505347160.c00015_PlaYM79...region1) | antiSMASH v5.0 | 4 | 2 |
| 8 *Paenibacillus peoriae* IBSD35 | [2816336711.Ga0347712_134.region1](https://img.jgi.doe.gov/cgi-bin/abc/main.cgi?section=BiosyntheticDetail&page=cluster_detail&cluster_id=2816336711.Ga0347712_134.region1) | antiSMASH v5.0 | 4 | 1 |
| 9 *Crocosphaera watsonii* WH 0003 | [2503285070.c00236_CWat_WH...region1](https://img.jgi.doe.gov/cgi-bin/abc/main.cgi?section=BiosyntheticDetail&page=cluster_detail&cluster_id=2503285070.c00236_CWat_WH...region1) | antiSMASH v5.0 | 5 | 6 |
| 10 *Bacillus cereus* RCH_BC2 | [2503539988.c00407_bcer176...region1](https://img.jgi.doe.gov/cgi-bin/abc/main.cgi?section=BiosyntheticDetail&page=cluster_detail&cluster_id=2503539988.c00407_bcer176...region1) | antiSMASH v5.0 | 4 | 3 |
| 11 *Pseudomonas aeruginosa* AMC, ATCC 142 | [2505315872.c00424_PaeATCC...region1](https://img.jgi.doe.gov/cgi-bin/abc/main.cgi?section=BiosyntheticDetail&page=cluster_detail&cluster_id=2505315872.c00424_PaeATCC...region1) | antiSMASH v5.0 | 4 | 2 |


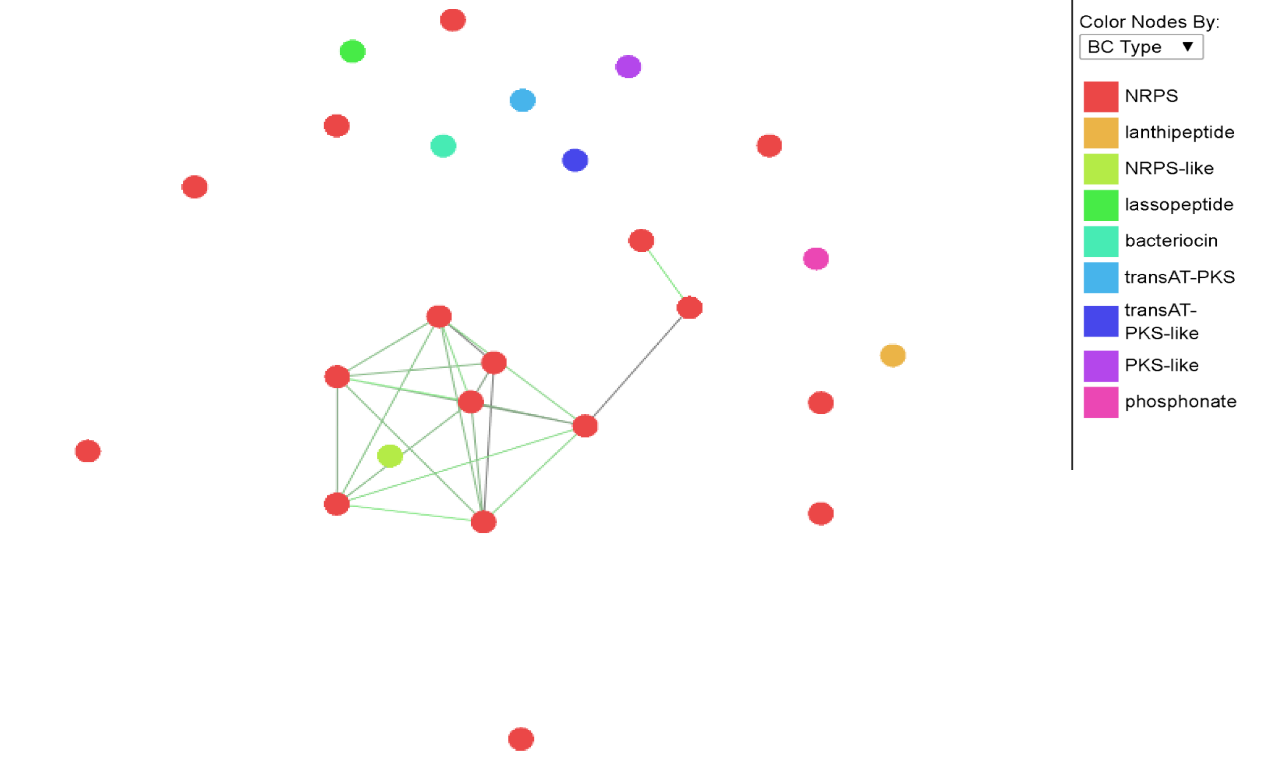


**FIGURE S1 |** 25 BGCs of *Paenibacillus peoriae* IBSD35 predicted by IMG/ABC web server, 18 BGCs are NRPS. BGC types are shown in colour codes. The most abundant BGC is NRPS (Red).


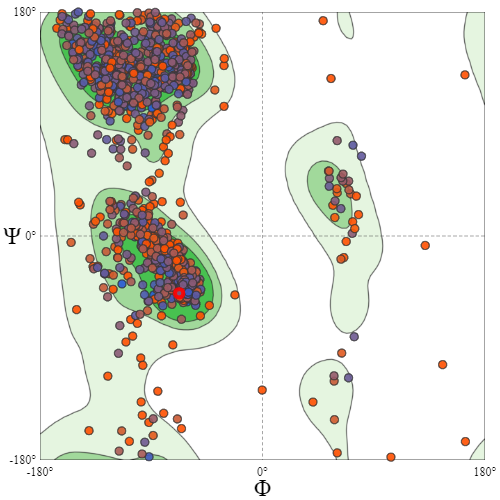


**FIGURE S2 |** The general Ramachandran plot and the Cβ deviation image for NRPS BGC. The ϕ, ψ values for each residue are plotted on a background of the smoothed contours. Over 90 % lie inside the inner favoured. Ramachandran outliers is 2.36%, Rotamer outliers is 1.17%, C-Beta deviation is 19, Bad Bond is 1/13627, Bad angles is 120/18507, Cis-non proline is 3/1615, Cis-prolines is 3/82, Twisted prolines is 3/82. Molprobity score is 1.59 and Clash score is 2.26. The Gly, Pro and pre-Pro residues are on separate plots (not shown).


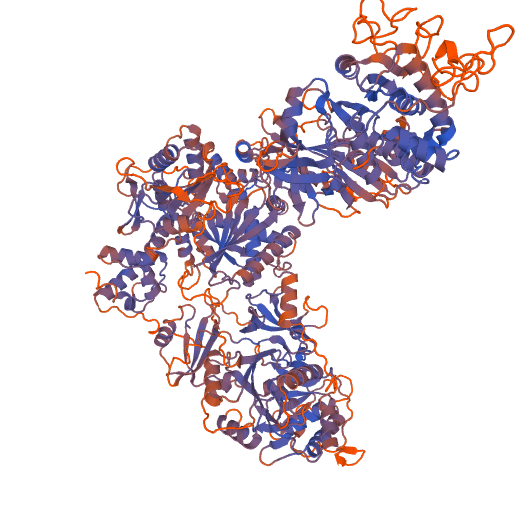


**FIGURE S3 |** The 3-D conformation model of an NRPS predicted from LC-MS data with an accession no. [A0A2S6P0H9](https://www.uniprot.org/uniprot/A0A2S6P0H9). It is generated from the SWISS-MODEL template library searched with BLAST and HHBlits and deposited at ModelArchive with accession no. [ma-z4hip](https://modelarchive.org/doi/10.5452/ma-z4hip).

**LINKS**

1. The 3-D conformation of the identified peptide (MESEDHISCLPYTNHVSRSTTVT SLNSHTYTLTFPTEISQR) was predicted using SWISS MODEL homology modelling and deposited in ModelArchive [ma-3rxzx](https://modelarchive.org/doi/10.5452/ma-3rxzx) and its NRPS 3D conformation model deposited at ModelArchive with accession no. maz4hip.

(DOI Will be activated as soon as the references or the article is added which refer to this ModelArchive.)

2. The data from [*Paenibacillus peoriae* IBSD35](https://www.ncbi.nlm.nih.gov/nuccore/NZ_PTJM00000000.1) whole genome project were submitted to EMBL/GenBank/DDBJ databases under BioProject; [PRJNA434168](https://www.ncbi.nlm.nih.gov/bioproject/PRJNA434168) and BioSample; [SAMN08537703](https://www.ncbi.nlm.nih.gov/biosample/SAMN08537703) and the GenBank accession number [PTJM01000000](https://www.ebi.ac.uk/ena/data/view/PTJM01000000).

3. Annotation was added by the NCBI Prokaryotic Genome Annotation Pipeline (released 2013). Information about the Pipeline can be found here:

<https://www.ncbi.nlm.nih.gov/genome/annotation_prok/>

4. JGI/ABC of the genome [*https://img.jgi.doe.gov/cgi-bin/abc/main.cgi?section=TaxonDetail&taxon_oid=2816332187*](https://img.jgi.doe.gov/cgi-bin/abc/main.cgi?section=TaxonDetail&taxon_oid=2816332187)

[*https://img.jgi.doe.gov/abc/antiSMASHv5/2816332187/index.html#*](https://img.jgi.doe.gov/abc/antiSMASHv5/2816332187/index.html)

5. The sequence shown here is derived from an EMBL/GenBank/DDBJ whole genome shotgun (WGS) entry which is a preliminary data with accession number [PPQ4949.1](https://www.ebi.ac.uk/ena/browser/view/PPQ49498).

[*https://antismash.secondarymetabolites.org/upload/bacteria-12cf44a5-576a-46d2-8ea6-503da4fa87fa/PTJM01000042.1.region001.gbk*](https://antismash.secondarymetabolites.org/upload/bacteria-12cf44a5-576a-46d2-8ea6-503da4fa87fa/PTJM01000042.1.region001.gbk) [AntiSMASH v5.0](https://antismash.secondarymetabolites.org/upload/bacteria-12cf44a5-576a-46d2-8ea6-503da4fa87fa/index.html#r19c1)

6. AntiSMASH predicted it is an NRPS and 60% of genes show similarity to Paenibacterin. <https://antismash.secondarymetabolites.org/upload/bacteria-12cf44a5-576a-46d2-8ea6-503da4fa87fa/index.html#r19c1>

7. Non-ribosomal peptide synthetase - <https://www.uniprot.org/uniprot/A0A2S6P0H9>

8. Secondary metabolites Biosynthetic gene clusters of *Paenibacillus peoriae* IBSD35 [antiSMASH v5.0](https://img.jgi.doe.gov/abc/antiSMASHv5/2816332187/index.html#r1c1)

9. Physiochemical properties of the AMPs were analyzed using ExPASy–ProtParam tool (<http://web.expasy.org/protparam>).

10. The NRPS predicted from LC-MS is 82.4% similarity to fusaricidin synthetase of *Paenibacillus* *polymyxa* SC2. ([E3EJA7](https://www.uniprot.org/uniprot/E3EJA7)).

11. LC-MS predicted a protein NRPS with accession number [A0A2S6P0H9](https://www.uniprot.org/uniprot/A0A2S6P0H9) and its gene is C5G87_06145.

12. Similar Clusters were search on the database already available in the JGI/ABC database and the Heatmap of 11 genomes were generated. <https://img.jgi.doe.gov/cgi-bin/abc/main.cgi>.
